# Influence of the Molecular Structure of Constituents and Liquid Phase Non-Ideality on the Viscosity of Deep Eutectic Solvents

**DOI:** 10.3390/molecules26144208

**Published:** 2021-07-11

**Authors:** Ahmad Alhadid, Liudmila Mokrushina, Mirjana Minceva

**Affiliations:** 1 Biothermodynamics, TUM School of Life Sciences, Technical University of Munich, 85354 Freising, Germany; ahmad.alhadid@tum.de; 2 Separation Science & Technology, Friedrich-Alexander-Universität Erlangen-Nürnberg (FAU), 91058 Erlangen, Germany; liudmila.mokrushina@fau.de

**Keywords:** hydrophobic deep eutectic solvents, solid–liquid equilibria, glass transition, monocarboxylic acids, terpenes

## Abstract

Hydrophobic deep eutectic solvents (DES) have recently been used as green alternatives to conventional solvents in several applications. In addition to their tunable melting temperature, the viscosity of DES can be optimized by selecting the constituents and molar ratio. This study examined the viscosity of 14 eutectic systems formed by natural substances over a wide range of temperatures and compositions. The eutectic systems in this study were classified as ideal or non-ideal based on their solid–liquid equilibria (SLE) data found in the literature. The eutectic systems containing constituents with cyclohexyl rings were considerably more viscous than those containing linear or phenyl constituents. Moreover, the viscosity of non-ideal eutectic systems was higher than that of ideal eutectic systems because of the strong intermolecular interactions in the liquid solution. At temperatures considerably lower than the melting temperature of the pure constituents, non-ideal and ideal eutectic systems with cyclohexyl constituents exhibited considerably high viscosity, justifying the kinetic limitations in crystallization observed in these systems. Overall, understanding the correlation between the molecular structure of constituents, SLE, and the viscosity of the eutectic systems will help in designing new, low-viscosity DES.

## 1. Introduction

Increasing awareness of environmental protection and process safety has led to green and sustainable chemistry as a separate research field [1]. One of the leading areas of research in green chemistry is green solvents [2]. Both ionic liquids (IL) and deep eutectic solvents (DES) can be considered green solvents depending on the toxicity and sustainability of their constituents [3,4]. DES may be better alternatives to IL because they can be formed by mixing inexpensive, biodegradable, and natural chemical substances [5,6]. Recently, the use of hydrophobic DES for extracting pollutants or target compounds from aqueous media has attracted intense research attention [7,8,9,10].

DES have a considerably higher viscosity than conventional solvents [11]. High viscosity increases the solvent flow, heat, and mass transfer resistance [12]. Therefore, low-viscosity DES are preferred to increase the extraction efficiency and decrease the energy demand for pumping and mixing the solvent in various process applications [13]. To decrease the viscosity, hydrophilic DES are usually diluted with water, but this is impossible for hydrophobic DES. The viscosity of several hydrophobic DES is available in the literature [6,14,15,16,17]. However, the existing data are only for mixtures at a specific molar ratio, usually at or close to the eutectic point. In most of the literature, the viscosity of DES was studied and discussed by treating eutectic mixtures of constituents with a fixed composition as a pseudo-compound without considering the effects of the constituents or their molar ratio [11,15,18,19,20,21].

This study examined how the molecular structure of the constituents and the intermolecular interactions in the liquid phase influence the viscosity of eutectic systems. The viscosities of 14 eutectic systems prepared by mixing terpenes and monocarboxylic acids over a wide range of temperatures and compositions were measured. To the best of our knowledge, the present work is the first to systematically measure the viscosity of eutectic systems over the whole composition range at which the mixture is liquid at room temperature. The eutectic systems were selected based on two criteria. First, the constituents have moderate melting temperatures to allow an easy comparison of their individual viscosities and the viscosity of the eutectic systems. Second, the solid–liquid equilibria (SLE) data of the systems are available in the literature to allow the intermolecular interactions in the liquid phase to be assessed and the systems classified as ideal or non-ideal [22,23,24]. It worth mentioning that SLE data are available only for eight systems. However, due to the similar chemical functionality of the constituents in the rest of binary eutectic systems, it is not expected that the solution behavior is different. The additional systems were considered—despite the absence of measured SLE data—because the aim of the work to systematically study the effect of the molecular structure of constituents on the viscosity of the eutectic system. 

## 2. Results

### 2.1. Viscosity of Pure Substances

**Figure 1:**
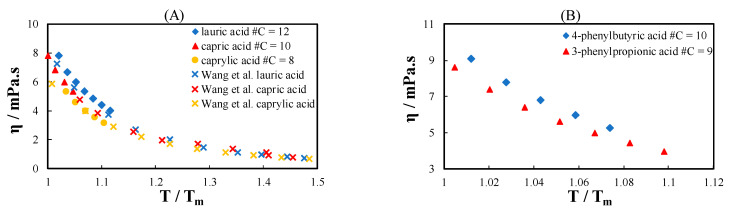

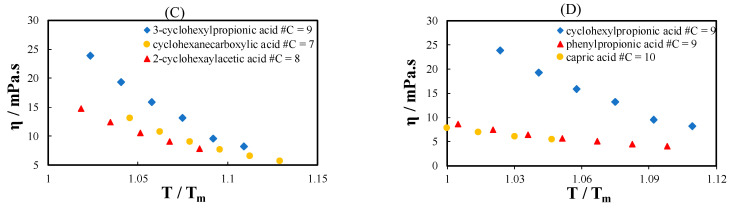
Viscosity of (**A**) linear acids, (**B**) phenyl acids, (**C**) cyclohexyl acids, and (**D**) acids with a similar number of carbon atoms with different hydrocarbon side chains as a function of temperature divided by the melting temperature of the pure acid. Filled symbols are data measured in this work, and cross symbols in (**A**) are data in the literature from Wang et al. [26].

As eutectic system constituents, three terpenes and nine monocarboxylic acids with different hydrocarbon side chains (linear, phenyl, or cyclohexyl) were selected. Their viscosity was determined at temperatures between 298.15 K and 353.15 K. The results are reported in Tables S1–S4 in Supplementary Materials. Figure 1 presents the viscosity of the acids measured in this work compared with the data in the literature as a function of the scaled temperature (T/T_m_). The viscosity data measured in this study are in good agreement with the data in the literature (Figure 1A). The viscosity of the linear acids increases with increasing number of carbon atoms in the hydrocarbon chain. The same tendency is observed for the phenyl and cyclohexyl acids (excluding the first member of the series), as shown in Figure 1B,C. However, the difference in viscosity of all acids is only apparent close to the melting temperature of the pure acids and converges near T/T_m_ = 1.1 to the same value. Acids with cyclohexyl rings possess very high viscosity near their melting temperature (Figure 1C). The higher viscosity of the chemical substances containing cyclohexyl rings can be attributed to interlocking between molecules [25]. Cyclohexyl rings are three dimensional, which increases the possibility of interlocking, particularly near the melting temperature.

Figure 1D shows the viscosity of acids from three different groups with a similar number of carbon atoms and different hydrocarbon side chains. The viscosities of 3-phenylpropionic acid and capric acid converge at low T/T_m_. Acids with cyclohexyl rings have a considerably higher viscosity than phenyl and linear acids. The viscosity of the linear, phenyl, and cyclohexyl acids tends to converge at a high T/T_m_. 

**Figure 2:**
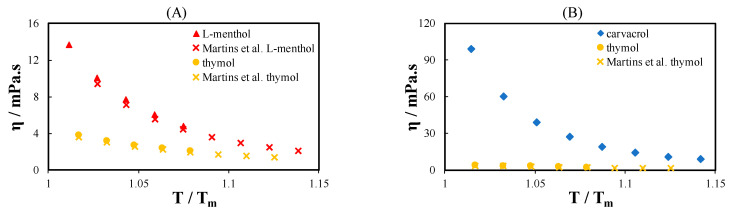
(**A**) Viscosity of l-menthol and thymol and (**B**) viscosity of thymol and carvacrol as a function of temperature normalized to the melting temperature of the pure terpenes. Filled symbols are data measured in this work, and cross symbols are data in the literature from Martins et al. [32].

Figure 2 shows the viscosity of the terpenes measured in this work compared with data from the literature. As shown in Figure 2A, the measured viscosity data are in good agreement with the data in the literature. In Figure 2A, the viscosity of the two terpenes with the same number of carbon atoms but different ring types is compared. The terpene with a cyclohexyl ring (l-Menthol) is more viscous than the terpene with a phenyl ring (thymol). The viscosity of the two terpenes tends to converge at high T/T_m_, similar to that observed for acids (Figure 1D). Figure 2B shows the viscosity of the two isomers: carvacrol and thymol. The difference between carvacrol and thymol viscosity near their melting temperatures is significant because of the significantly lower melting temperature of carvacrol (T_m_ = 274.2 K) than of thymol (T_m_ = 322.7 K). 

Overall, substances containing cyclohexyl rings are generally more viscous than linear and phenyl substances. Likewise, the same trend was observed with hydrocarbons, in which the viscosity order was cyclohexane > benzene > n-hexane [27,28,29], in high molecular weight hydrocarbons [30], and ester liquid crystal components [31].

### 2.2. Viscosity of Eutectic Systems

**Figure 3:**
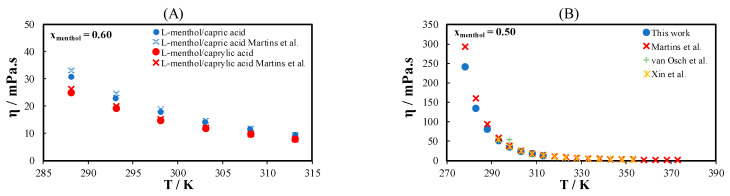
(**A**) Viscosity of l-menthol/caprylic acids and l-menthol/capric acid and (**B**) viscosity of l-menthol/thymol measured in this study compared with the data in the literature. Filled symbols are data measured in this work, and cross symbols are data in the literature from Martins et al. [16,32], van Osch et al. [5], and Xin et al. [33].

The viscosities of 14 binary eutectic systems containing terpenes and monocarboxylic acids were measured at temperatures between 278.15 and 313.15 K over the composition range in which the mixture is liquid at room temperature. The experimental data are reported in Tables S5–S18 in Supplementary Materials. Out of the 14 binary eutectic systems studied in this work, data on the viscosity of only four eutectic systems at or near the eutectic composition are available in the literature [5,16,33]. Figure 3 compares the viscosity measured in this work with those found in the literature for l-menthol/thymol (MTH), l-menthol/caprylic acid, and l-menthol/capric acid. The experimental data are in good agreement with the data in the literature, except for the temperature range below 290 K. The history of the sample before the viscosity measurements may be a reason for this discrepancy. Differences in the viscosity were observed between samples left for a long time at low temperatures without agitation and those stirred at high shear rates using a magnetic stirrer immediately before the start of the viscosity measurement.

Based on the data in the literature on SLE, the binary eutectic systems studied were classified as either ideal systems, i.e., the intermolecular interactions are similar to those in the pure liquid state, or non-ideal systems, i.e., with strong intermolecular interactions between unlike molecules. Figure S1 in the Supporting Information presents the solid–liquid phase diagrams and the deviation from the ideal liquidus lines of the studied systems. 

**Figure 4:**
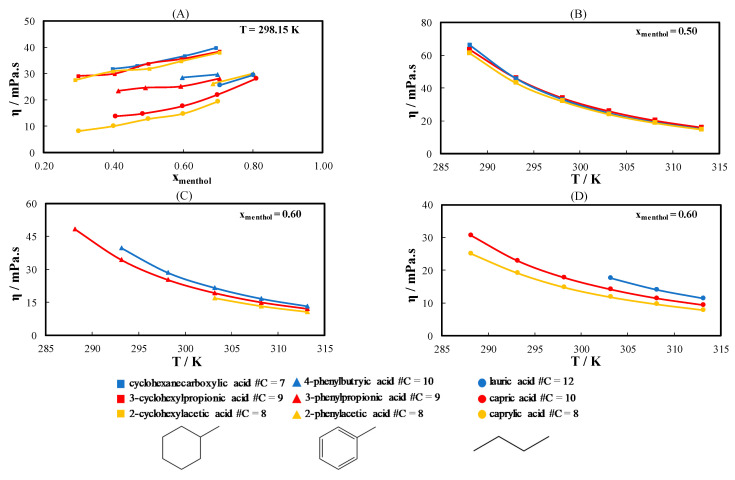
(**A**) Viscosity of ideal eutectic systems containing l-menthol and different monocarboxylic acids as a function of the l-menthol composition at T = 298.15 K. (**B**) Viscosity of the eutectic systems containing l-menthol and different cyclohexyl acids at x_menthol_ = 0.50. (**C**) Viscosity of the eutectic systems containing l-menthol and different phenyl acids at x_menthol_ = 0.60. (**D**) Viscosity of the eutectic systems containing l-menthol and different linear acids at x_menthol_ = 0.60.

Figure 4 shows the measured viscosity data of nine ideal eutectic systems composed of l-menthol and several monocarboxylic acids at different temperatures. As shown in Figure 4A, the viscosity of the eutectic systems at 298.15 K increases with increasing amount of l-menthol in the mixture. The viscosity of the eutectic systems containing cyclohexyl acids is higher than those containing phenyl or linear acids. The two previous observations are attributed directly to the viscosity of pure constituents. l-menthol has a higher viscosity than monocarboxylic acids, and cyclohexyl acids are more viscous than linear and phenyl acids. The increase in the viscosity of the mixture due to the increase in the amount of l-menthol in the mixtures is more pronounced in the eutectic systems containing linear acids than in the systems containing cyclic acids, which can be justified by the lower viscosity of the linear acids than of the cyclic acids. As noticed in Figure 4A, the viscosity of the eutectic systems containing cyclohexyl acids is similar regardless of the number of carbon atoms in the acid.

Figure 4B–D presents the viscosity of the eutectic systems containing l-menthol and different cyclohexyl, phenyl, and linear acids near the eutectic composition of the mixtures (see Table 2) as a function of temperature. The three eutectic systems formed by cyclohexyl acids have similar viscosities (Figure 4B) independent of the chain length of the cyclohexyl acid. In contrast, increasing the chain length of the linear and phenyl acids increases the viscosity of the eutectic system, as shown in Figure 4C,D, respectively. For linear and phenyl acids, as depicted in Figure 4C,D, the difference in the viscosity of the systems formed by acids with a different number of carbon atoms increases at low temperatures. 

**Figure 5:**
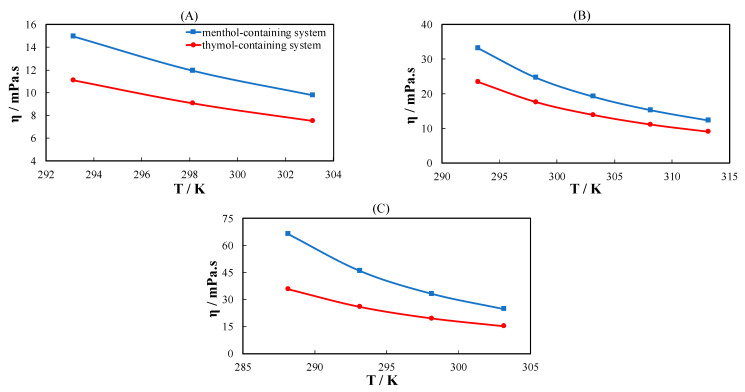
Viscosity of ideal eutectic systems containing either l-menthol or thymol with (**A**) caprylic acid (linear), (**B**) 3-phenylpropionic acid (phenyl), or (**C**) cyclohexanecarboxylic acid (cyclohexyl) with an equimolar ratio as a function of temperature.

Figure 5 shows the measured viscosity of two more ideal eutectic systems formed by mixing thymol or l-menthol with caprylic acid (Figure 5A), 3-phenylpropionic acid (Figure 5B), and cyclohexanecarboxylic acid (Figure 5C) at an equimolar ratio between the constituents. This ratio is close to the eutectic composition of these eutectic systems calculated using the ideal solution model. As shown in Figure 5, the eutectic systems containing l-menthol have a higher viscosity than the eutectic systems containing thymol. This is because l-menthol contains a cyclohexyl ring, whereas thymol contains a phenyl ring. The difference in viscosity between ideal eutectic systems containing thymol or l-menthol increases in the order cyclohexyl > phenyl > linear and is more pronounced at lower temperatures. Generally, the trends observed for all studied ideal systems were the same as in the case of the pure substances.

**Figure 6:**
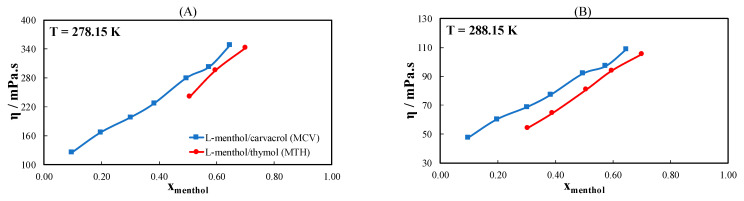

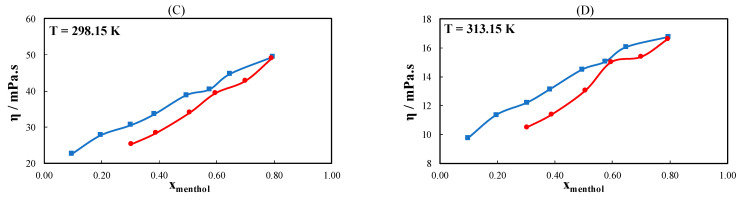
Viscosity of binary eutectic systems containing l-menthol with thymol or carvacrol as a function of l-menthol composition at (**A**) T = 278.15 K, (**B**) T = 288.15 K, (**C**) T = 298.15 K, and (**D**) T = 313.15 K.

The results for the two non-ideal systems, namely, MTH and l-menthol/carvacrol (MCV), are shown in Figure 6 at T = 278.15 (Figure 6A), 288.15 (Figure 6B), 298.15 (Figure 6C), and 313.15 K (Figure 6D) as a function of the l-menthol composition. The two eutectic systems had a eutectic composition and temperature of x_e, menthol_ = 0.57 and 0.42 and T_e_ = 241.5 K and 243.3 K for MTH and MCV, respectively. As shown in Figure 6, the viscosity of MCV is higher than that of MTH, which can be attributed to the higher viscosity of carvacrol (see Figure 2B). At all four temperatures, a slight increase in the viscosity of the MTH system is observed near its eutectic composition. This can be explained by the fact that in non-ideal eutectic systems, the intermolecular interactions are strongest near the eutectic point of the system. However, this is not observed in MCV. This can be justified by the lesser negative deviation from ideal behavior observed in MCV compared to MTH [24].

A previous study [24] reported that MCV, MTH, l-menthol/3-cyclohexylpropionic acid, and l-menthol/cyclohexanecarboxylic acid formed glassy phases upon crystallization. As shown in Figure 4A and Figure 6A, these systems have very high viscosity, which can explain the observed kinetic limitation during crystallization [34]. The high viscosity and the glass formation can result from strong intermolecular interactions, the low melting temperature of the mixture, and the molecular structure of the constituents, i.e., cyclohexyl ring. The latter explains why aqueous sugar solutions, i.e., sugars containing saturated rings [35,36,37], and borneol and camphor based eutectic systems [38] are glass-forming mixtures. 

**Figure 7:**
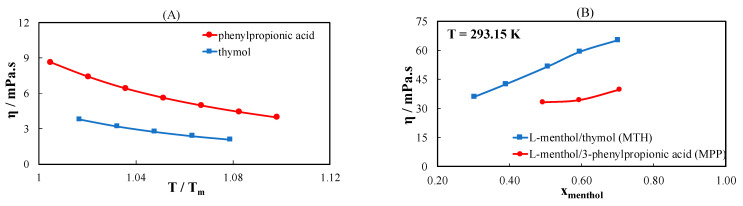

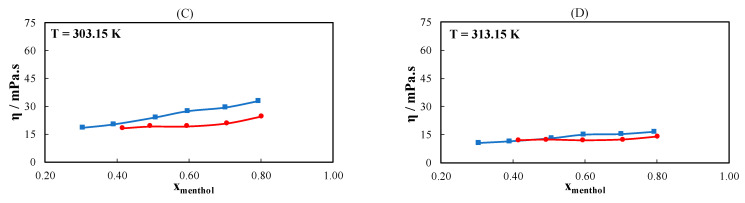
(**A**) Viscosity of thymol and 3-phenylpropionic acid as a function of the scaled temperature. (**B**) Viscosity of the eutectic systems containing l-menthol with thymol or 3-phenylpropionic acid as a function of the l-menthol composition at (**B**) T = 293.15 K, (**C**) T = 303.15 K, and (**D**) T = 313.15 K.

The influence of the intermolecular interaction on the viscosity was examined by comparing the viscosity of l-menthol/3-phenylpropionic acid (MPP) (ideal eutectic system) with MTH (non-ideal eutectic system) at different temperatures and l-menthol compositions. Figure 7A presents the measured viscosity of pure 3-phenylpropionic acid and thymol. Despite containing a phenyl ring and having similar melting temperatures, 3-phenylpropionic acid is more viscous than thymol. Figure 7B–D compares the viscosities of their mixtures with l-menthol at T = 293.15 K, 303.15 K, and 313.15 K, respectively, as a function of the l-menthol composition. Although the viscosity of pure thymol is lower than that of 3-phenylpropionic acid, the viscosity of MTH is considerably higher than that of MPP over the entire l-menthol composition range and at all temperatures (Figure 7B–D). This is attributed to the strong intermolecular interactions in non-ideal eutectic system, i.e., MTH. The viscosity of both systems increases with increasing amount of l-menthol in the mixture. Moreover, the difference between the viscosities of both systems decreases at high temperatures. This obviously suggests that the effect of intermolecular interactions is pronounced at low temperatures. At 313.15 K (Figure 7D), the viscosity of both systems converged to the same value. The difference in viscosity between MTH and MPP is insignificant for the mixtures with a small amount of l-menthol in the mixture. In summary, the non-ideal eutectic system is more viscous than the ideal eutectic system.

## 3. Materials and Methods

Table 1 lists the chemical substances used to prepare the eutectic systems along with their molecular structure, source, declared purity, water content, and melting temperature. The substances were used as received. The water content was measured using Karl-Fischer coulometric titrator (Hanna Instrument, Woonsocket, RI, USA). The eutectic systems were prepared by weighing the constituents (precision $1\times 10^{-4}$ g; Sartorius, Goettingen, Germany) in glass vessels at various ratios. The vessels were then closed, and the mixtures were heated gently under continuous stirring until a homogenous clear liquid was obtained. Table 2 lists the eutectic systems analyzed in this study, which were classified based on the available SLE diagrams as ideal and non-ideal. The eutectic compositions and eutectic temperatures of those were taken from [23,24,32]. If the experimental eutectic composition and temperature are equal to or close to those calculated using the ideal solution model, the system is ideal. On the contrary, a substantial difference between the experimental values and calculated values is observed for non-ideal eutectic systems. The SLE phase diagrams of the systems can be found in Figure S1 in Supplementary Materials.

The viscosity of the pure substances and mixtures was measured using an Anton Paar MCR301 rheometer (Anton Paar GmbH, Austria) with a 50 mm diameter cone-plate geometry (CP-50) and a 0.101 mm gap. The mixtures were then applied to the lower plate using a pipette. The lower plate was in contact with Peltier system, providing temperature control to ±0.01 K. Five minutes was sufficient to achieve thermal equilibrium. The measurements were taken at temperatures between 298.15 K and 353.15 K. At first, the viscosity was measured at variable shear rates from 1 to 100 s^−1^. In all samples, a Newtonian behavior was observed. The viscosity measured at variable shear rates of selected systems is shown in Figures S2–S4 in Supplementary Materials. The viscosity was then measured at a constant shear rate of 10 s^−1^, and the data are reported as the average of six points after 180 s of continuous shearing. The average standard deviation of the measured viscosity values is 2.6%. The temperature dependence of the viscosity was modeled using Vogel-Fulcher-Tammann (VFT) equation as follows [40]:

(1) $$\ln\eta =A+\frac{B}{T-C}$$

The obtained parameters (A, B, and C) for pure constituents and the eutectic systems and the absolute average deviation (AAD) between experimental and calculated viscosities are available in Tables S19–S33 in Supplementary Materials.

## 4. Conclusions

This study examined the effects of the viscosity of the individual constituents and the intermolecular interactions on the viscosity of the eutectic system. The viscosity of 14 hydrophobic eutectic systems as well as of 12 their constituents was measured at different temperatures using a rheometer. For the mixtures, the measurements were carried out over the composition range in which the mixture is liquid at room temperature. 

The viscosity of pure constituents correlated with their molecular structure, and the viscosity of pure acids and alcohols studied in this work was observed in the following order: cyclohexyl > phenyl > linear. The viscosity of all substances studied tends to converge at T/T_m_ > 1.2.

Based on the available SLE diagrams, the eutectic systems were classified as ideal and non-ideal. The ideal eutectic systems prepared by linear and phenyl constituents have a considerably lower viscosity than the eutectic systems prepared from constituents containing a cyclohexyl ring, in accordance with the trends observed for the pure substances. In the case of the non-ideal systems, the strong intermolecular interactions between unlike molecules make an additional effect on the viscosity of the mixture. The effects of both the intermolecular interactions and the molecular structure of constituents are pronounced at temperatures significantly lower than the melting temperature of the constituents. Therefore, glass formation can be attributed to the high viscosity of the non-ideal eutectic systems and eutectic systems containing constituents with cyclohexyl rings.

At high temperatures, intermolecular interactions weaken, and the viscosity of the pure constituents has a major influence. As a result, the difference between the viscosity of the ideal and non-ideal eutectic systems examined in this study decreased at 313.15 K. In general, the viscosity of pure constituents, ideal and non-ideal eutectic systems was observed to converge at high temperatures. 

Although substantial negative deviation from ideal behavior is preferred in DES to have a larger liquid window, the viscosity of the non-ideal eutectic systems would be significantly high and, thus, are unfavorable in applications. Therefore, understanding the interplay between the non-ideality of DES and the structure of DES constituents is essential for designing low-viscosity hydrophobic DES.

## Figures and Tables

**Table 1:** Substances used to prepare the eutectic mixtures along with their structure, source, purity, water content, and melting temperature (T_m_).

Name	Structure	Supplier	Declared Purity/%	Water Content/mg·g^−1^	T_m_/K
l-Menthol	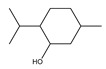	Sigma Aldrich	≥99	0.1075	314.6 [23]
Thymol	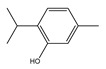	Sigma Aldrich	>99	0.0869	322.7 [24]
Carvacrol	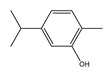	Sigma Aldrich	>99	5.9269	274.2 [24]
Caprylic acid	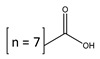	Merck	99	1.2530	288.0 [23]
Capric acid	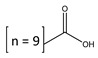	Alfa Aesar	99	0.1670	303.9 [23]
Lauric acid	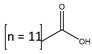	Merck	99	0.0842	316.6 [23]
2-Phenylacetic acid	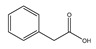	Alfa Aesar	≥99	0.0656	349.2 [39]
3-Phenylpropionic acid	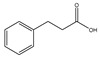	Alfa Aesar	99	0.0504	321.6 [23]
4-Phenylbutyric acid	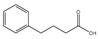	Alfa Aesar	>99	0.0779	324.2 [39]
Cyclohexanecarboxylic acid	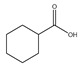	ThermoFisher	98	2.2280	299.4 [23]
2-Cyclohexylacetic acid	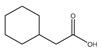	Alfa Aesar	≥98	0.7425	302.6 [39]
3-Cyclohexylpropionic acid	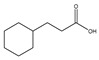	ThermoFisher	>98	1.2636	291.3 [23]

**Table 2:** Eutectic systems studied in this work classified according to the behavior of components in the liquid phase along with their experimental and calculated eutectic composition (x_e_) and eutectic temperature (T_e_) assuming ideal solution model.

Constituents	x_e, menthol_		T_e_/K	
	Experimental	Calculated *	Experimental	Calculated *
Ideal eutectic systems				
l-Menthol + caprylic acid	0.50 [23]	0.42	266.7 [23]	270.1
l-Menthol + capric acid	0.60 [23]	0.56	279.7 [23]	283.2
l-Menthol + lauric acid	0.70 [23]	0.67	289.5 [23]	292.5
l-Menthol + 2-phenylacetic acid	–	0.65	290.8	–
l-Menthol + 3-phenylpropionic acid	0.60 [23]	0.55	283.1 [23]	282.1
l-Menthol + 4-phenylbutyric acid	–	0.61	287.2	–
l-Menthol + cyclohexanecarboxylic acid	0.50 [23]	0.40	265.0 [23]	267.7
l-Menthol + 2-cyclohexylacetic acid	–	0.45	272.9	–
l-Menthol + 3-cyclohexylpropionic acid	0.50 [23]	0.42	262.1 [23]	270.2
Thymol + caprylic acid	0.35 [32]	0.29	275.0 [32]	277.5
Thymol + 3-phenylpropionic acid	–	0.44	291.6	–
Thymol + cyclohexanecarboxylic acid	–	0.29	277.6	–
Non-ideal eutectic systems				
l-Menthol + thymol	0.57 [24]	0.61	241.5 [24]	287.5
l-Menthol + carvacrol	0.42 [24]	0.30	243.3 [24]	256.0

* Calculated using the ideal solution model.

## Data Availability

Data are available on request.

## Supplementary Materials

The following are available online. Figure S1. Solid–liquid phase diagram of eutectic systems formed by mixing l-menthol with (A) 3-cyclohexylpropionic acid, (B) caprylic acid, (C) cyclohexanecarboxylic acid, (D) capric acid, (E) 3-phenylpropionic acid, (F) lauric acid, (G) thymol, and (H) carvacrol. Figure S2. Viscosity of l-menthol/capric acid system measured at variable shear rates. Figure S3. Viscosity of l-menthol/2-phenylacetic acid system measured at variable shear rates. Figure S4. Viscosity of l-menthol/3-cyclohexylpropionic acid system measured at variable shear rates. Figure S5. Viscosity of l-menthol/thymol system measured at variable shear rates. Table S1. Viscosity of linear acids measured in this work. Table S2. Viscosity of phenyl acids measured in this work. Table S3. Viscosity of cyclohexyl acids measured in this work. Table S4. Viscosity of terpenes measured in this work. Table S5. Viscosity of l-menthol/caprylic acid. Table S6. Viscosity of l-menthol/capric acid. Table S7. Viscosity of l-menthol/lauric acid. Table S8. Viscosity of l-menthol/cyclohexanecarboxylic acid. Table S9. Viscosity of l-menthol/2-cyclohexylacetic acid. Table S10. Viscosity of l-menthol/3-cyclohexylpropionic acid. Table S11. Viscosity of l-menthol/2-phenylacetic acid. Table S12. Viscosity of l-menthol/3-phenylpropionic acid. Table S13. Viscosity of l-menthol/4-phenylbutyric acid. Table S14. Viscosity of thymol/cyclohexanecarboxylic acid. Table S15. Viscosity of thymol/caprylic acid. Table S16. Viscosity of thymol/3-phenylpropionic acid. Table S17. Viscosity of l-menthol/carvacrol. Table S18. Viscosity of l-menthol/thymol. Table S19. Vogel-Fulcher-Tammann parameters and the absolute average deviation (AAD) between experimental and calculated viscosity of pure constituents. Table S20. Vogel-Fulcher-Tammann parameters and the absolute average deviation (AAD) between experimental and calculated viscosity of l-menthol/caprylic acid eutectic system. Table S21. Vogel-Fulcher-Tammann parameters and the absolute average deviation (AAD) between experimental and calculated viscosity of l-menthol/capric acid eutectic system. Table S22. Vogel-Fulcher-Tammann parameters and the absolute average deviation (AAD) between experimental and calculated viscosity of l-menthol/lauric acid eutectic system. Table S23. Vogel-Fulcher-Tammann parameters and the absolute average deviation (AAD) between experimental and calculated viscosity of l-menthol/2-phenylacetic acid eutectic system. Table S24. Vogel-Fulcher-Tammann parameters and the absolute average deviation (AAD) between experimental and calculated viscosity of l-menthol/3-phenylpropionic acid eutectic system. Table S25. Vogel-Fulcher-Tammann parameters and the absolute average deviation (AAD) between experimental and calculated viscosity of l-menthol/4-phenylbutyric acid eutectic system. Table S26. Vogel-Fulcher-Tammann parameters and the absolute average deviation (AAD) between experimental and calculated viscosity of l-menthol/cyclohexanecarboxylic acid eutectic system. Table S27. Vogel-Fulcher-Tammann parameters and the absolute average deviation (AAD) between experimental and calculated viscosity of l-menthol/2-cyclohexylacetic acid eutectic system. Table S28. Vogel-Fulcher-Tammann parameters and the absolute average deviation (AAD) between experimental and calculated viscosity of l-menthol/3-cyclohexylpropionic acid eutectic system. Table S29. Vogel-Fulcher-Tammann parameters and the absolute average deviation (AAD) between experimental and calculated viscosity of thymol/caprylic acid eutectic system. Table S30. Vogel-Fulcher-Tammann parameters and the absolute average deviation (AAD) between experimental and calculated viscosity of thymol/cyclohexanecarboxylic acid eutectic system. Table S31. Vogel-Fulcher-Tammann parameters and the absolute average deviation (AAD) between experimental and calculated viscosity of thymol/3-phenylpropionic acid eutectic system. Table S32. Vogel-Fulcher-Tammann parameters and the absolute average deviation (AAD) between experimental and calculated viscosity of l-menthol/thymol eutectic system. Table S33. Vogel-Fulcher-Tammann parameters and the absolute average deviation (AAD) between experimental and calculated viscosity of l-menthol/carvacrol eutectic system.
