# Supplementary material for: Influence of the Molecular Structure of Constituents and Liquid Phase Non-Ideality on the Viscosity of Deep Eutectic Solvents
Source: Molecules. 2021 Jul 11;26(14):4208. doi: 10.3390/molecules26144208 (PMC8308104; doi:10.3390/molecules26144208)
Supplement: Supplementary file 1 [file molecules-26-04208-s001.zip › molecules-1298711-supplementary.pdf]

Supplementary Materials

Influence of the molecular structure of constituents and liquid phase non-ideality on the viscosity of deep eutectic solvents

Ahmad Alhadid [1], Liudmila Mokrushina [2], and Mirjana Minceva* [1]

[1] Biothermodynamics, TUM School of Life Sciences, Technical University of Munich, Germany

[2] Separation Science & Technology, Friedrich-Alexander-Universität Erlangen-Nürnberg (FAU), Germany

*Corresponding author e-mail: mirjana.minceva@tum.de

Table S1. Viscosity of linear acids measured in this work

T	caprylic acid	capric acid	lauric acid
298.15 K	5.30 ± 0.02	–	–
303.15 K	4.53 ± 0.02	7.77 ± 0.05	–
308.15 K	3.96 ± 0.02	6.78 ± 0.05	–
313.15 K	3.50 ± 0.01	5.99 ± 0.05	–
318.15 K	3.11 ± 0.01	5.30 ± 0.05	–
323.15 K	–	–	7.77 ± 0.13
328.15 K	–	–	6.67 ± 0.08
333.15 K	–	–	6.97 ± 0.08
338.15 K	–	–	5.33 ± 0.04
343.15 K	–	–	4.83 ± 0.07
348.15 K	–	–	4.39 ± 0.08
353.15 K	–	–	3.99 ± 0.06

Table S2. Viscosity of phenyl acids measured in this work

T	3-phenylpropionic acid	4-phenylbutyric acid
323.15 K	8.64 ± 0.01	–
328.15 K	7.42 ± 0.01	9.09 ± 0.02
333.15 K	6.42 ± 0.01	7.82 ± 0.01
338.15 K	5.63 ± 0.02	6.82 ± 0.01
343.15 K	4.96 ± 0.01	5.98 ± 0.01
348.15 K	4.43 ± 0.02	5.28 ± 0.01
353.15 K	3.97 ± 0.01	–

Table S3. Viscosity of cyclohexyl acids measured in this work

T	cyclohexanecarboxylic acid	2-cyclohexylacetic acid	3-cyclohexylpropionic acid
298.15 K	–	–	23.90 ± 0.09
303.15 K	–	–	19.24 ± 0.14
308.15 K	–	14.76 ± 0.10	15.83 ± 0.11
313.15 K	13.09 ± 0.06	12.36 ± 0.11	13.20 ± 0.13
318.15 K	10.67 ± 0.05	10.57 ± 0.10	9.46 ± 0.12
323.15 K	8.91 ± 0.01	9.08 ± 0.05	8.22 ± 0.11
328.15 K	7.55 ± 0.02	7.88 ± 0.07	–
333.15 K	6.51 ± 0.01	–	–
338.15 K	5.66 ± 0.02	–	–

Table S4. Viscosity of terpenes measured in this work

T	L-menthol	thymol	carvacrol
278.15 K	–	–	98.74 ± 0.11
283.15 K	–	–	60.11 ± 0.03
288.15 K	–	–	39.12 ± 0.03
293.15 K	–	–	26.89 ± 0.06
298.15 K	–	–	19.16 ± 0.02
303.15 K	–	–	14.21 ± 0.02
308.15 K	–	–	10.92 ± 0.01
313.15 K	–	–	8.64 ± 0.03
318.15 K	13.70 ± 0.01	–	–
323.15 K	10.10 ± 0.01	–	–
328.15 K	7.76 ± 0.04	3.80 ± 0.01	–
333.15 K	6.08 ± 0.01	3.21 ± 0.02	–
338.15 K	4.85 ± 0.01	2.75 ± 0.01	–
343.15 K	–	2.39 ± 0.02	–
348.15 K	–	2.09 ± 0.01	–

Table S5. Viscosity of L-menthol/caprylic acid

T	$x_{menthol}$ = 0.30	$x_{menthol}$ = 0.40	$x_{menthol}$ = 0.49	$x_{menthol}$ = 0.60	$x_{menthol}$ = 0.70	$x_{menthol}$ = 0.80
288.15 K	–	–	21.63 ± 0.30	24.98 ± 0.08	35.03 ± 0.07	–
293.15 K	9.81 ± 0.01	12.29 ± 0.05	16.27 ± 0.07	19.07 ± 0.07	25.71 ± 0.05	–
298.15 K	8.22 ± 0.02	10.11 ± 0.04	12.75 ± 0.11	14.76 ± 0.05	19.34 ± 0.04	–
303.15 K	7.00 ± 0.04	8.37 ± 0.01	10.13 ± 0.10	11.77 ± 0.01	–	14.87 ± 0.04
308.15 K	5.99 ± 0.04	7.07 ± 0.02	8.20 ± 0.08	9.59 ± 0.07	11.72 ± 0.03	14.32 ± 0.03
313.15 K	5.17 ± 0.02	6.01 ± 0.03	6.81 ± 0.11	7.83 ± 0.03	9.38 ± 0.03	11.11 ± 0.03

Table S6. Viscosity of L-menthol/capric acid

T	$x_{menthol}$ = 0.41	$x_{menthol}$ = 0.48	$x_{menthol}$ = 0.60	$x_{menthol}$ = 0.70	$x_{menthol}$ = 0.81
288.15 K	–	23.85 ± 0.10	30.55 ± 0.03	–	–
293.15 K	17.17 ± 0.05	18.62 ± 0.03	22.81 ± 0.04	29.42 ± 0.08	–
298.15 K	13.84 ± 0.03	14.84 ± 0.03	17.68 ± 0.05	21.97 ± 0.02	28.13 ± 0.08
303.15 K	11.36 ± 0.01	12.08 ± 0.01	14.88 ± 0.01	16.88 ± 0.03	20.61 ± 0.03
308.15 K	9.50 ± 0.04	10.01 ± 0.03	11.34 ± 0.02	13.25 ± 0.05	15.61 ± 0.07
313.15 K	7.98 ± 0.02	8.38 ± 0.02	9.28 ± 0.02	10.59 ± 0.05	12.04 ± 0.05

Table S7. Viscosity of L-menthol/lauric acid

T	$x_{menthol}$ = 0.60	$x_{menthol}$ = 0.71	$x_{menthol}$ = 0.80
298.15 K	–	25.62 ± 0.04	29.57 ± 0.01
303.15 K	17.59 ± 0.03	19.40 ± 0.02	21.85 ± 0.03
308.15 K	14.01 ± 0.02	15.19 ± 0.01	16.63 ± 0.03
313.15 K	11.38 ± 0.02	12.15 ± 0.03	12.88 ± 0.02

Table S8. Viscosity of L-menthol/cyclohexanecarboxylic acid

T	$x_{menthol}$ = 0.40	$x_{menthol}$ = 0.47	$x_{menthol}$ = 0.60	$x_{menthol}$ = 0.70	$x_{menthol}$ = 0.77
288.15 K	59.84 ± 0.30	66.23 ± 0.13	78.22 ± 0.11	–	–
293.15 K	43.59 ± 0.03	45.77 ± 0.14	52.31 ± 0.05	57.63 ± 0.09	–
298.15 K	31.89 ± 0.06	33.08 ± 0.14	36.75 ± 0.08	39.72 ± 0.08	–
303.15 K	24.30 ± 0.08	24.65 ± 0.14	26.87 ± 0.10	28.30 ± 0.09	31.63 ± 0.23
308.15 K	19.97 ± 0.08	19.00 ± 0.12	20.29 ± 0.07	20.97 ± 0.06	22.74 ± 0.07
313.15 K	15.16 ± 0.10	14.89 ± 0.09	15.70 ± 0.24	16.11 ± 0.23	17.12 ± 0.13

Table S9. Viscosity of L-menthol/2-cyclohexylacetic acid

T	$x_{menthol}$ = 0.29	$x_{menthol}$ = 0.40	$x_{menthol}$ = 0.50	$x_{menthol}$ = 0.60	$x_{menthol}$ = 0.70	$x_{menthol}$ = 0.79
288.15 K	47.87 ± 0.15	55.82 ± 0.24	61.28 ± 0.20	70.76 ± 1.2	–	–
293.15 K	35.77 ± 0.15	40.65 ± 0.16	43.04 ± 0.09	48.45 ± 0.65	53.23 ± 0.89	–
298.15 K	27.48 ± 0.16	30.80 ± 0.51	31.84 ± 0.15	34.68 ± 0.28	38.00 ± 0.16	–
303.15 K	21.67 ± 0.12	23.34 ± 0.06	23.98 ± 0.12	25.90 ± 0.28	27.39 ± 0.14	28.70 ± 0.10
308.15 K	17.30 ± 0.13	18.38 ± 0.02	18.66 ± 0.05	20.12 ± 0.63	20.41 ± 0.13	20.78 ± 0.04
313.15 K	14.02 ± 0.07	14.91 ± 0.24	14.67 ± 0.09	15.29 ± 0.32	15.57 ± 0.11	15.76 ± 0.08

Table S10. Viscosity of L-menthol/3-cyclohexylpropionic acid

T	$x_{menthol}$ = 0.30	$x_{menthol}$ = 0.40	$x_{menthol}$ = 0.50	$x_{menthol}$ = 0.60	$x_{menthol}$ = 0.70	$x_{menthol}$ = 0.80
288.15 K	50.52 ± 0.25	54.41 ± 0.02	63.37 ± 0.24	71.12 ± 1.4	–	–
293.15 K	37.67 ± 0.06	39.55 ± 0.03	45.83 ± 0.18	49.98 ± 0.28	54.63 ± 0.36	–
298.15 K	29.06 ± 0.05	29.97 ± 0.06	33.78 ± 0.04	35.51 ± 0.07	38.34 ± 0.07	–
303.15 K	22.85 ± 0.05	23.22 ± 0.05	25.78 ± 0.10	26.76 ± 0.18	27.82 ± 0.08	30.22 ± 0.08
308.15 K	18.36 ± 0.09	18.44 ± 0.06	20.05 ± 0.08	20.44 ± 0.07	20.85 ± 0.11	21.94 ± 0.05
313.15 K	14.97 ± 0.10	14.84 ± 0.06	15.79 ± 0.05	15.97 ± 0.05	15.95 ± 0.05	16.45 ± 0.07

Table S11. Viscosity of L-menthol/2-phenylacetic acid

T	$x_{menthol}$ = 0.59	$x_{menthol}$ = 0.69	$x_{menthol}$ = 0.80
298.15 K	–	26.11 ± 0.12	30.08 ± 0.27
303.15 K	16.98 ± 0.08	19.33 ± 0.01	20.45 ± 0.03
308.15 K	13.22 ± 0.01	14.80 ± 0.02	15.21 ± 0.02
313.15 K	10.53 ± 0.01	11.57 ± 0.03	11.69 ± 0.08

Table S12. Viscosity of L-menthol/3-phenylpropionic acid

T	$x_{menthol}$ = 0.41	$x_{menthol}$ = 0.49	$x_{menthol}$ = 0.59	$x_{menthol}$ = 0.70	$x_{menthol}$ = 0.80
288.15 K	–	–	48.43 ± 0.07	–	–
293.15 K	–	33.18 ± 0.21	34.33 ± 0.10	39.69 ± 0.03	–
298.15 K	23.56 ± 0.06	24.72 ± 0.23	25.21 ± 0.10	28.14 ± 0.01	–
303.15 K	18.26 ± 0.09	19.18 ± 0.32	19.19 ± 0.13	20.79 ± 0.02	24.38 ± 0.07
308.15 K	14.52 ± 0.10	15.28 ± 0.33	14.90 ± 0.02	15.85 ± 0.01	18.12 ± 0.10
313.15 K	11.93 ± 0.12	12.31 ± 0.27	11.92 ± 0.03	12.33 ± 0.01	13.82 ± 0.13

Table S13. Viscosity of L-menthol/4-phenylbutyric acid

T	$x_{menthol}$ = 0.42	$x_{menthol}$ = 0.49	$x_{menthol}$ = 0.60	$x_{menthol}$ = 0.70	$x_{menthol}$ = 0.79
293.15 K	–	–	39.82 ± 0.11	–	–
298.15 K	–	–	28.47 ± 0.04	29.67 ± 0.17	–
303.15 K	–	20.40 ± 0.29	21.58 ± 0.05	22.15 ± 0.38	25.80 ± 0.35
308.15 K	17.41 ± 0.13	16.42 ± 0.89	16.59 ± 0.07	16.73 ± 0.19	18.32 ± 0.01
313.15 K	13.93 ± 0.10	12.73 ± 0.13	13.11 ± 0.13	12.94 ± 0.18	14.27 ± 0.05

Table S14. Viscosity of thymol/cyclohexanecarboxylic acid

T	x_{thymol} = 0.40	x_{thymol} = 0.50	x_{thymol} = 0.60
288.15 K	38.19 ± 0.07	35.61 ± 0.10	34.92 ± 0.10
293.15 K	28.75 ± 1.4	25.79 ± 0.07	25.21± 0.13
298.15 K	21.55 ± 0.59	19.45 ± 0.06	18.89± 0.11
303.15 K	16.67 ± 0.12	15.12 ± 0.05	14.59± 0.06

Table S15. Viscosity of thymol/caprylic acid

T	x_{thymol} = 0.30	x_{thymol} = 0.49	x_{thymol} = 0.50
288.15 K	10.56 ± 0.01	12.02 ± 0.02	13.84 ± 0.06
293.15 K	8.72 ± 0.02	9.71 ± 0.02	11.11 ± 0.03
298.15 K	7.38 ± 0.04	8.10 ± 0.05	9.07 ± 0.03
303.15 K	6.29 ± 0.01	6.82 ± 0.02	7.51 ± 0.02

Table S16. Viscosity of thymol/3-phenylpropionic acid

T	x_{thymol} = 0.51	x_{thymol} = 0.62
293.15 K	23.37 ± 0.30	–
298.15 K	17.63 ± 0.05	–
303.15 K	13.89 ± 0.12	13.58 ± 0.08
308.15 K	11.13 ± 0.05	10.83 ± 0.05
313.15 K	9.07 ± 0.05	8.77 ± 0.08

Table S17. Viscosity of L-menthol/carvacrol

T	$x_{menthol}$ = 0.10	$x_{menthol}$ = 0.20	$x_{menthol}$ = 0.30	$x_{menthol}$ = 0.38	$x_{menthol}$ = 0.49	$x_{menthol}$ = 0.57	$x_{menthol}$ = 0.65	$x_{menthol}$ = 0.80
278.15 K	125.06 ± 0.18	166.43 ± 0.20	198.12 ± 0.25	226.74 ± 0.08	279.40 ± 0.19	302.53 ± 0.21	347.46 ± 0.39	–
283.15 K	74.33 ± 0.03	96.70 ± 0.09	112.53 ± 0.03	127.44 ± 0.03	154.64 ± 0.02	164.85 ± 0.05	187.05 ± 0.04	–
288.15 K	47.42 ± 0.04	60.20 ± 0.02	68.8 ± 0.08	77.15 ± 0.06	91.88 ± 0.01	97.18 ± 0.09	108.90 ± 0.04	–
293.15 K	32.06 ± 0.02	39.97 ± 0.03	44.75 ± 0.04	49.63 ± 0.05	58.23 ± 0.03	61.05 ± 0.03	68.08 ± 0.06	–
298.15 K	22.65 ± 0.03	27.75 ± 0.04	30.56 ± 0.03	33.54 ± 0.02	38.84 ± 0.03	40.41 ± 0.02	44.71 ± 0.03	49.39 ± 0.04
303.15 K	16.59 ± 0.03	19.89 ± 0.03	21.76 ± 0.03	23.73 ± 0.03	27.09 ± 0.03	28.03 ± 0.03	30.62 ± 0.02	33.00 ± 0.02
308.15 K	12.52 ± 0.01	14.87 ± 0.03	16.01 ± 0.04	17.34 ± 0.04	19.51 ± 0.04	20.19 ± 0.01	21.83 ± 0.05	23.13 ± 0.02
313.15 K	9.74 ± 0.04	11.36 ± 0.01	12.20 ± 0.05	13.14 ± 0.03	14.50 ± 0.03	15.04 ± 0.04	16.05 ± 0.04	16.77 ± 0.01

Table S18. Viscosity of L-menthol/thymol

T	$x_{menthol}$ = 0.30	$x_{menthol}$ = 0.39	$x_{menthol}$ = 0.51	$x_{menthol}$ = 0.60	$x_{menthol}$ = 0.70	$x_{menthol}$ = 0.79
278.15 K	–	–	241.63 ± 0.09	296.08 ± 0.53	342.64 ± 0.27	–
283.15 K	–	105.16 ± 0.13	134.71 ± 0.15	160.52 ± 0.29	182.66 ± 0.06	–
288.15 K	54.20 ± 0.06	64.55 ± 0.04	80.85 ± 0.07	94.06 ± 0.21	105.51 ± 0.08	–
293.15 K	36.08 ± 0.05	42.49 ± 0.08	51.73 ± 0.05	59.38 ± 0.53	65.36 ± 0.03	–
298.15 K	25.26 ± 0.04	28.35 ± 0.04	33.97 ± 0.03	39.33 ± 0.38	42.71 ± 0.06	48.96 ± 0.01
303.15 K	18.45 ± 0.06	20.26 ± 0.02	24.01 ± 0.03	27.37 ± 0.32	29.32 ± 0.05	32.76 ± 0.03
308.15 K	13.69 ± 0.01	15.01 ± 0.03	17.47 ± 0.03	20.11 ± 0.53	20.89 ± 0.01	23.00 ± 0.04
313.15 K	10.49 ± 0.03	11.38 ± 0.02	13.05 ± 0.03	15.01 ± 0.39	15.39 ± 0.02	16.63 ± 0.01

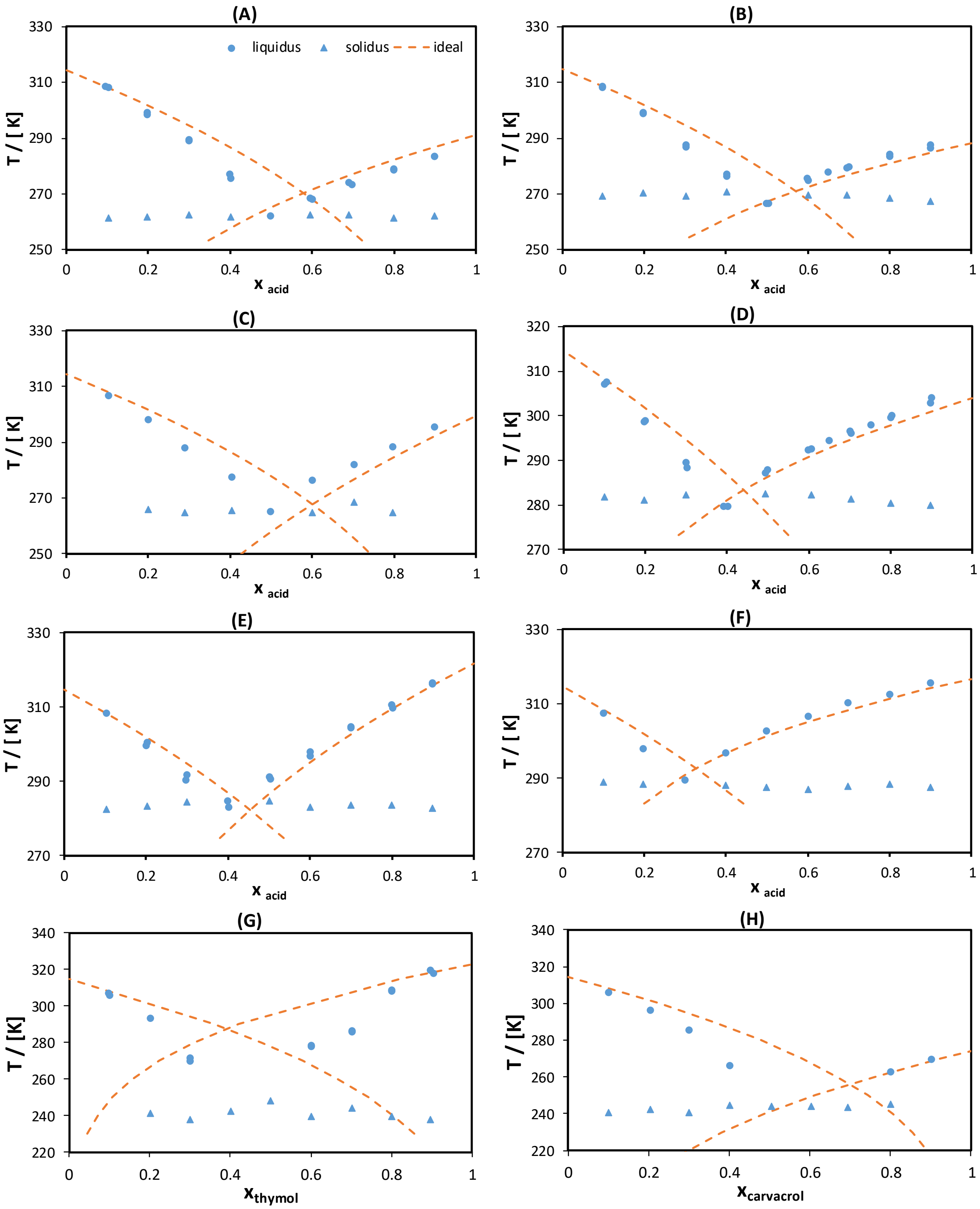

Figure S1. Solid–liquid phase diagram of eutectic systems formed by mixing L-menthol with (A) 3-cyclohexylpropionic acid, (B) caprylic acid, (C) cyclohexanecarboxylic acid, (D) capric acid, (E) 3-phenylpropionic acid, (F) lauric acid, (G) thymol, and (H) carvacrol. Data were taken from Alhadid et al. [1,2]

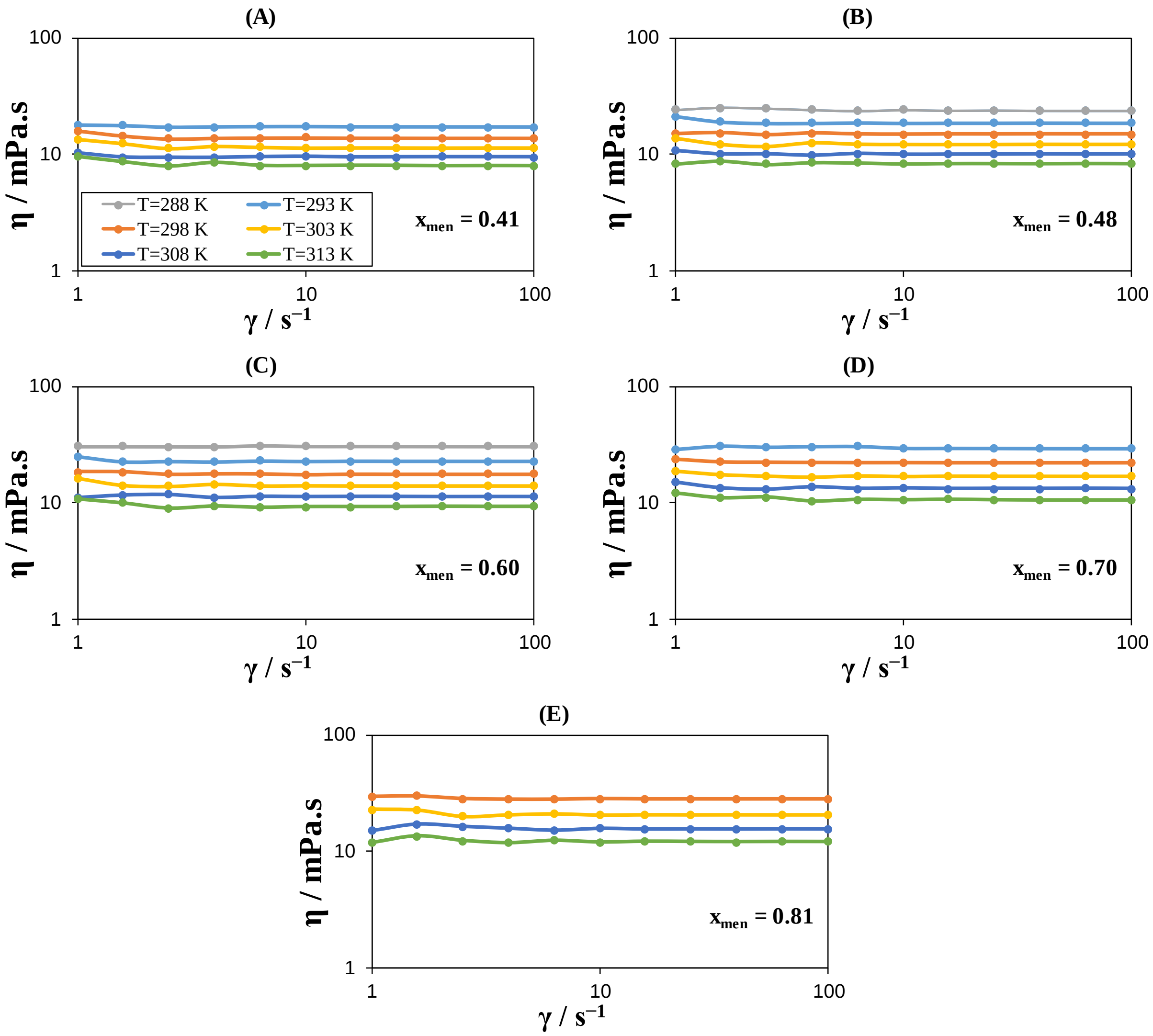

Figure S2. Viscosity of L-menthol/capric acid system measured at variable shear rates.

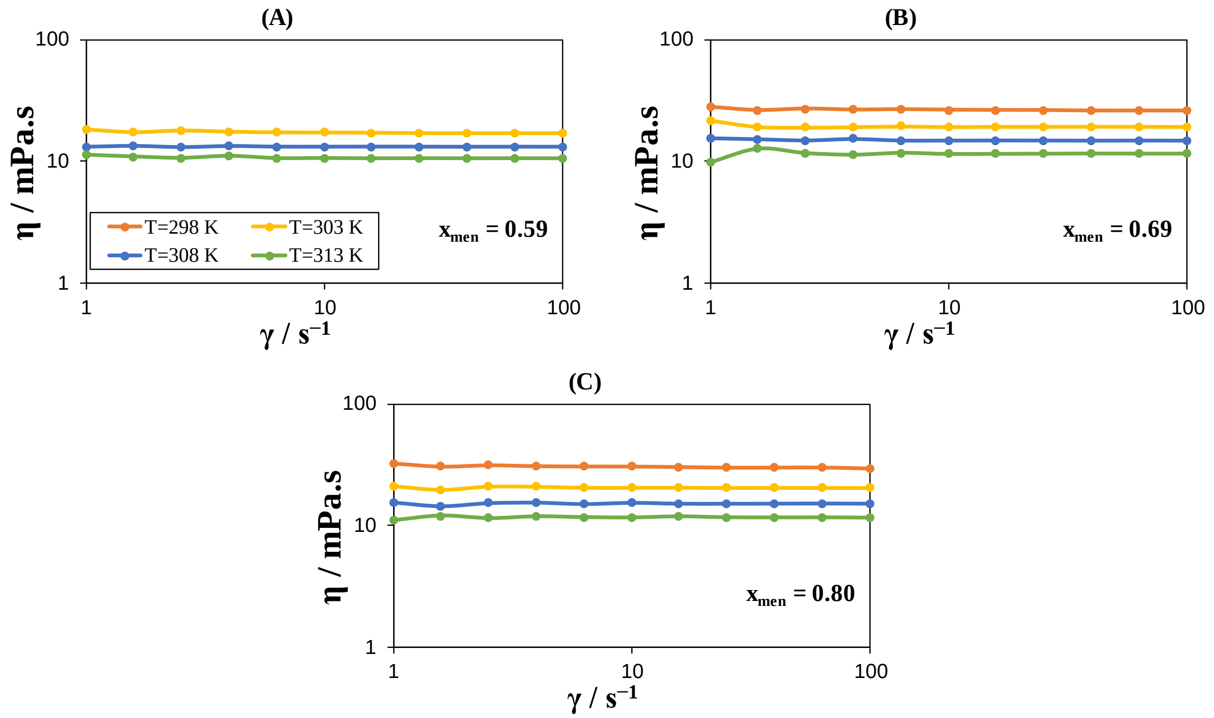

Figure S3. Viscosity of L-menthol/2-phenylacetic acid system measured at variable shear rates.

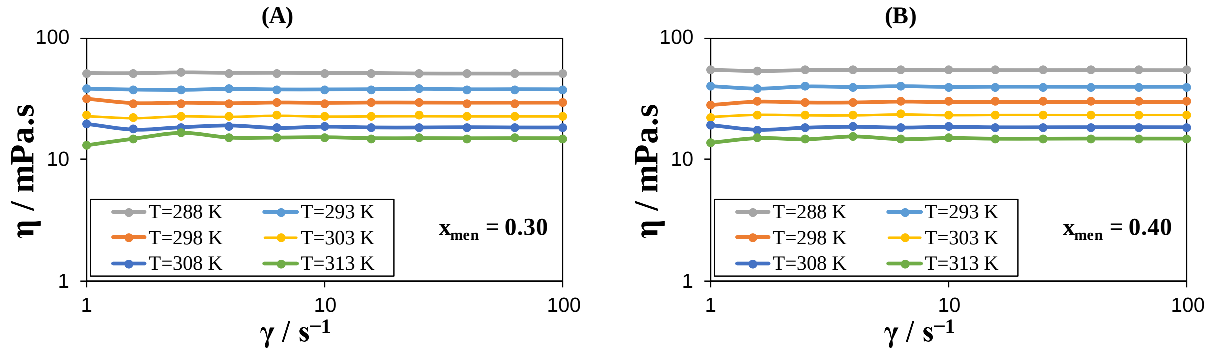

Figure S4. Viscosity of L-menthol /3-cyclohexylpropionic acid system measured at variable shear rates.

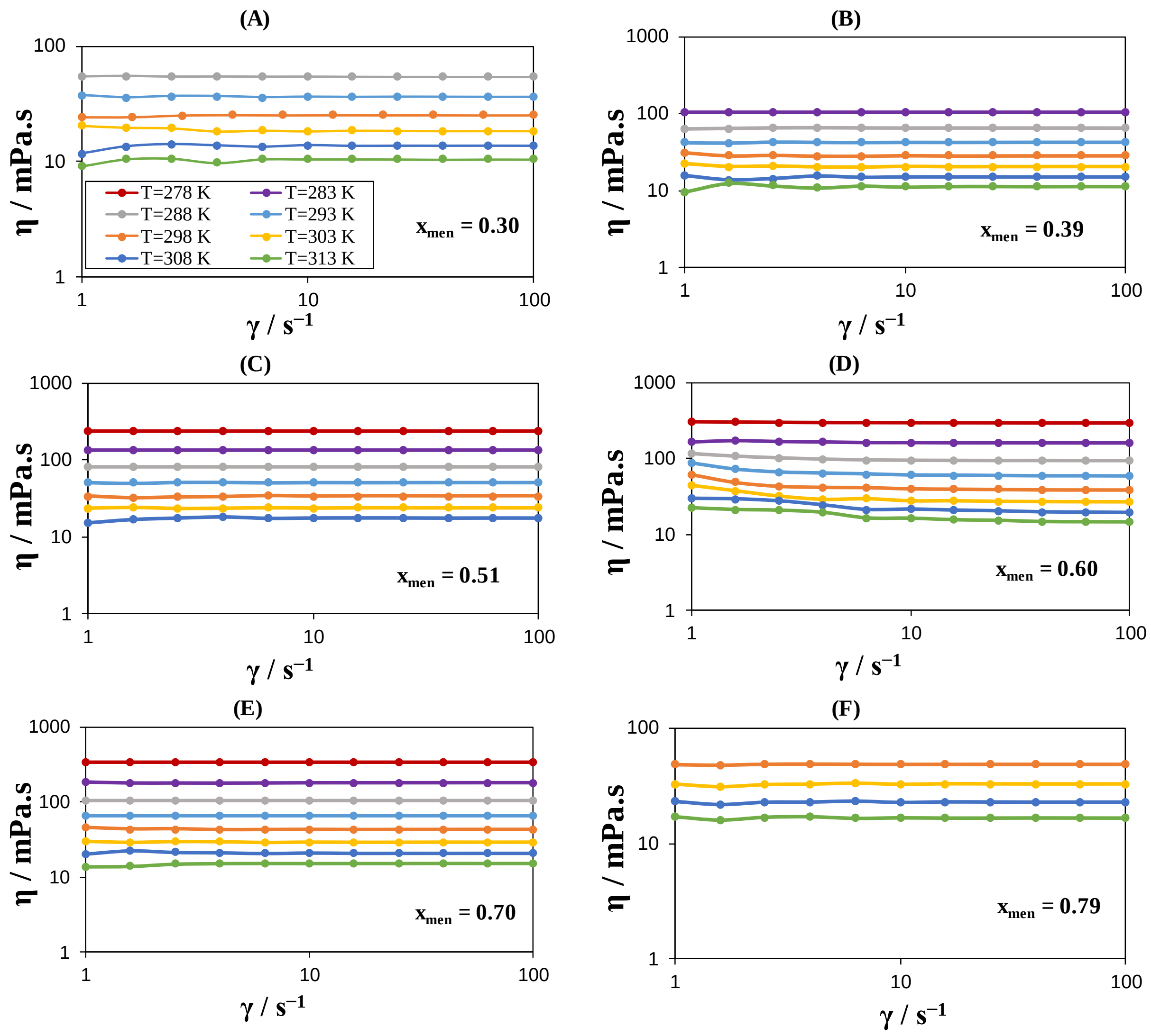

Figure S5. Viscosity of L-menthol/thymol system measured at variable shear rates.

Table S19. Vogel-Fulcher-Tammann parameters and the absolute average deviation (AAD) between experimental and calculated viscosity of pure constituents.

Substance	A	B	C	AAD / mPa.s
L-Menthol	−2.8426	467.3746	232.5409	0.04
Thymol	–2.7399	473.7001	211.9264	0.01
Carvacrol	–2.4499	464.8747	212.1361	0.03
Caprylic acid	–1.1096	304.1467	190.2933	0.04
Capric acid	–1.8553	540.7565	164.7126	0.01
Lauric acid	–0.4747	215.5802	237.6622	0.03
3-phenylpropionic acid	–1.9845	538.6208	193.0829	0.01
4-phenylbutyric acid	–2.1322	607.7107	188.1123	0.01
Cyclohexanecarboxylic acid	–1.7659	456.1377	207.8333	0.01
2-Cyclohexylacetic acid	–1.8614	571.5703	182.6063	0.02
3-Cyclohexylpropionic acid	–6.1637	1852.633	100.0002	0.42

Table S20. Vogel-Fulcher-Tammann parameters and the absolute average deviation (AAD) between experimental and calculated viscosity of L-menthol/caprylic acid eutectic system.

L-Menthol mole fraction	A	B	C	AAD / mPa.s
x_{men} = 0.10	−3.2275	989.2448	100	0.04
x_{men} = 0.20	–2.6021	689.0243	146.5482	0.01
x_{men} = 0.30	–3.4350	907.8471	134.3775	0.01
x_{men} = 0.40	–3.0828	762.0987	156.8644	0.01
x_{men} = 0.50	–1.8129	433.1696	197.2895	0.01
x_{men} = 0.60	–3.8941	911.1977	159.96	0.06
x_{men} = 0.70	–3.5028	768.1734	179.332	0.01
x_{men} = 0.80	–0.0102	224.968	220.1228	0.01

Table S21. Vogel-Fulcher-Tammann parameters and the absolute average deviation (AAD) between experimental and calculated viscosity of L-menthol/capric acid eutectic system.

L-Menthol mole fraction	A	B	C	AAD / mPa.s
x_{men} = 0.41	–2.3432	601.1032	177.2365	0.01
x_{men} = 0.48	–2.1185	537.7202	186.5115	0.01
x_{men} = 0.60	–1.9071	465.8010	200.6883	0.05
x_{men} = 0.70	–2.8105	627.6134	191.7937	0.01
x_{men} = 0.81	–3.0381	623.9595	200.2695	0.02

Table S22. Vogel-Fulcher-Tammann parameters and the absolute average deviation (AAD) between experimental and calculated viscosity of L-menthol/lauric acid eutectic system.

L-Menthol mole fraction	A	B	C	AAD / mPa.s
x_{men} = 0.60	–2.2445	548.4842	195.8606	0.01
x_{men} = 0.71	–1.5593	392.1857	216.4891	0.01
x_{men} = 0.80	–3.4154	734.1679	190.2140	0.02

Table S23. Vogel-Fulcher-Tammann parameters and the absolute average deviation (AAD) between experimental and calculated viscosity of L-menthol/2-phenylacetic acid eutectic system.

L-Menthol mole fraction	A	B	C	AAD / mPa.s
x_{men} = 0.59	–2.7703	599.7928	196.091	0.01
x_{men} = 0.69	–2.3506	497.3947	209.535	0.01
x_{men} = 0.80	–0.0629	139.7480	257.834	0.02

Table S24. Vogel-Fulcher-Tammann parameters and the absolute average deviation (AAD) between experimental and calculated viscosity of L-menthol/3-phenylpropionic acid eutectic system.

L-Menthol mole fraction	A	B	C	AAD / mPa.s
x_{men} = 0.41	–0.8358	291.1428	225.28	0.02
x_{men} = 0.49	–1.9871	498.1523	202.4	0.03
x_{men} = 0.59	–2.1689	500.8480	205.354	0.02
x_{men} = 0.70	–2.2138	478.0371	212.0540	0.03
x_{men} = 0.80	–3.2768	673.38	199.0812	0.01

Table S25. Vogel-Fulcher-Tammann parameters and the absolute average deviation (AAD) between experimental and calculated viscosity of L-menthol/4-phenylbutyric acid eutectic system.

L-Menthol mole fraction	A	B	C	AAD / mPa.s
x_{men} = 0.49	–6.8548	2006.1	100	0.17
x_{men} = 0.60	–1.4405	372.971	220.360	0.09
x_{men} = 0.70	–6.4726	1606.5821	135.261	0.02
x_{men} = 0.79	1.0642	58.8281	276.24	0.01

Table S26. Vogel-Fulcher-Tammann parameters and the absolute average deviation (AAD) between experimental and calculated viscosity of L-menthol/cyclohexanecarboxylic acid eutectic system.

L-Menthol mole fraction	A	B	C	AAD / mPa.s
x_{men} = 0.40	–1.7028	467.540	207.600	0.22
x_{men} = 0.47	–2.0311	494.8467	208.64	0.03
x_{men} = 0.60	–2.0062	472.7572	213.884	0.02
x_{men} = 0.69	–2.4819	537.3941	210.934	0.06
x_{men} = 0.77	–0.9113	266.9086	242.0101	0.01

Table S27. Vogel-Fulcher-Tammann parameters and the absolute average deviation (AAD) between experimental and calculated viscosity of L-menthol/2-cyclohexylacetic acid eutectic system.

L-Menthol mole fraction	A	B	C	AAD / mPa.s
x_{men} = 0.29	–2.4550	658.667	183.9825	0.04
x_{men} = 0.40	–2.7285	689.440	186.0185	0.12
x_{men} = 0.50	–2.107	525.4687	203.6989	0.10
x_{men} = 0.59	–2.683	621.5403	198.5988	0.14
x_{men} = 0.70	–5.585	1289.295	158.3013	0.09
x_{men} = 0.79	–0.8059	247.343	243.734	0.01

Table S28. Vogel-Fulcher-Tammann parameters and the absolute average deviation (AAD) between experimental and calculated viscosity of L-menthol/3-cyclohexylpropionic acid eutectic system.

L-Menthol mole fraction	A	B	C	AAD / mPa.s
x_{men} = 0.30	–1.9224	557.647	192.7330	0.04
x_{men} = 0.40	–1.7457	493.7269	202.1613	0.07
x_{men} = 0.50	–3.2648	804.4826	179.6456	0.06
x_{men} = 0.59	–2.8586	669.3541	194.1928	0.15
x_{men} = 0.70	–3.0226	661.5476	198.9552	0.01
x_{men} = 0.80	–2.5975	533.2239	214.3715	0.01

Table S29. Vogel-Fulcher-Tammann parameters and the absolute average deviation (AAD) between experimental and calculated viscosity of thymol/caprylic acid eutectic system.

Thymol mole fraction	A	B	C	AAD / mPa.s
x_{thy} = 0.30	–1.4291	359.818	193.0988	0.01
x_{thy} = 0.40	–1.1200	291.2857	207.3682	0.01
x_{thy} = 0.50	–2.9397	677.3579	166.4808	0.01

Table S30. Vogel-Fulcher-Tammann parameters and the absolute average deviation (AAD) between experimental and calculated viscosity of thymol/cyclohexanecarboxylic acid eutectic system.

Thymol mole fraction	A	B	C	AAD / mPa.s
x_{thy} = 0.40	–7.5907	2113.764	100	0.09
x_{thy} = 0.50	–1.4097	360.3068	215.83	0.01
x_{thy} = 0.60	–1.9203	432.7841	209.0788	0.01

Table S31. Vogel-Fulcher-Tammann parameters and the absolute average deviation (AAD) between experimental and calculated viscosity of thymol/3-phenylpropionic acid eutectic system.

Thymol mole fraction	A	B	C	AAD / mPa.s
x_{thy} = 0.51	–1.4945	365.5468	209.459	0.04
x_{thy} = 0.62	1.0333	36.3352	281.2166	0.01

Table S32. Vogel-Fulcher-Tammann parameters and the absolute average deviation (AAD) between experimental and calculated viscosity of L-menthol/thymol eutectic system.

L-Menthol mole fraction	A	B	C	AAD / mPa.s
x_{men} = 0.30	–2.8754	549.2505	208.1738	0.06
x_{men} = 0.39	–3.2796	608.2821	206.4916	0.16
x_{men} = 0.51	–3.3404	623.2377	207.5504	0.13
x_{men} = 0.59	–2.5209	502.9973	216.9605	0.40
x_{men} = 0.70	–3.0790	584.7195	212.5884	0.15
x_{men} = 0.79	–4.6419	893.8334	193.223	0.17

Table S33. Vogel-Fulcher-Tammann parameters and the absolute average deviation (AAD) between experimental and calculated viscosity of L-menthol/carvacrol eutectic system.

L-Menthol mole fraction	A	B	C	AAD / mPa.s
x_{men} = 0.10	–2.4608	475.2950	212.9472	0.04
x_{men} = 0.20	–2.6558	517.1928	211.5908	0.06
x_{men} = 0.30	–2.7861	536.0183	211.7698	0.01
x_{men} = 0.38	–2.9199	562.3020	210.7583	0.02
x_{men} = 0.49	–3.0392	591.3891	209.9544	0.30
x_{men} = 0.57	–3.0752	591.9334	210.7886	0.02
x_{men} = 0.65	–3.1477	608.8155	210.4912	0.09
x_{men} = 0.79	–2.8664	534.7218	219.1214	0.01

References

Alhadid, A.; Mokrushina, L.; Minceva, M., Design of Deep Eutectic Systems: A Simple Approach for Preselecting Eutectic Mixture Constituents. *Molecules* **2020,** *25* (5), 1077.

Alhadid, A.; Mokrushina, L.; Minceva, M., Formation of glassy phases and polymorphism in deep eutectic solvents. *J. Mol. Liq.* **2020,** *314*, 113667.
